# Exploring women and traditional birth attendants’ perceptions and experiences of stillbirths in district Thatta, Sindh, Pakistan: a qualitative study

**DOI:** 10.1186/s12978-020-0852-0

**Published:** 2020-01-13

**Authors:** Sanam Zulfiqar Mcnojia, Sarah Saleem, Anam Feroz, Kausar S. Khan, Farnaz Naqvi, Shiyam Sunder Tikmani, Elizabeth M. McClure, Sameen Siddiqi, Robert L. Goldenberg

**Affiliations:** 1 Department of Community Health Sciences, The Aga Khan University, Stadium Road, PO Box 3500, Karachi, 74800 Pakistan; 2 RTI International, Durham, USA; 3 Department of Obstetrics and Gynecology, Columbia University, New York, USA

**Keywords:** Stillbirths, Rural setting, Qualitative study, Women perspectives, Traditional birth attendant’s perspectives

## Abstract

**Background:**

Pakistan reports the highest stillbirth rate in the world at 43 per thousand births with more than three-quarters occurring in rural areas. The Global Network for Women’s and Children’s Health maintains a Maternal and Newborn Health Registry (MNHR) in 14 study clusters of district Thatta, Sindh Pakistan. For the last 10 years, the MNHR has recorded a high stillbirths rate with a slow decline. This exploratory study was designed to understand the perspectives of women and traditional birth attendants regarding the high occurrence of stillbirth in Thatta district.

**Methods:**

We used an exploratory qualitative study design by conducting in-depth interviews (IDIs) and focus group discussions (FGDs) using semi-structured interview guide with rural women (FGDs = 4; *n* = 29) and traditional birth attendants (FGDs = 4; *n* = 14) who were permanent residents of Thatta. In addition, in-depth interviews were conducted with women who had experienced a stillbirth (IDIs = 4). This study presents perceptions and experiences of women and TBAs regarding high rate of stillbirth in Thatta district, Karachi.

**Results:**

Women showed reluctance to receive skilled/ standard care when in need due to apprehensions towards operative delivery, poor attitude of skilled health care providers, and poor quality of care as service delivery factors. High cost of care, far distance to facility, lack of transport and need of an escort from the family or village to visit a health facility were additional important factors for not seeking care resulting in stillbirth. The easy availability of unskilled provider in the form of traditional birth attendant is then preferred over a skilled health care provider. TBAs shared their husband or family members restrict them to visit or consult a doctor during pregnancy. According to TBAs after delivering a macerated fetus, women are given herbs to remove infection from woman‘s body and uterus. Further women are advised to conceive soon so that they get rid of infections.

**Conclusion:**

Women of this rural community carry lots of apprehension against skilled medical care and as a result follow traditional practices. Conscious efforts are required to increase the awareness of women to develop positive health seeking behavior during pregnancy, delivery and the post-partum period. Alongside, provision of respectful maternity care needs to be emphasized especially at public health facilities.

## Plain English language summary

In Pakistan, despite numerous initiatives for safe motherhood, no substantial decline has been observed either in maternal mortality or in stillbirths; the stillbirth rates remain high at 43 per 1000 births. The MNHR show high rates of stillbirths in rural district Thatta, Sindh, Pakistan. This study aimed to understand the perspectives of women and TBAs regarding the high stillbirth rate in rural district Thatta. The study employed an exploratory qualitative research design. The data collection method included in-depth interviews (IDIs) and focus group discussions (FGDs) using semi-structured interview guide. This study provided an insight into the views of women and traditional birth attendants regarding stillbirths. Women mentioned apprehensions towards operative delivery, poor attitude of skilled health care providers, poor quality of care as service delivery factors which make them not to access health facilities. TBAs shared that husbands restrict women to visit or consult doctors during pregnancy.

The study concluded that efforts are required to increase the awareness of women and their husbands to develop positive health seeking behavior. Alongside, provision of respectful maternity care needs to be emphasized especially at public health facilities.

## Background

World Health Organization (WHO) (2016) defines stillbirth as “baby born with no signs of life” [1]. The lower gestational age limit varies by location from 20 to 28 weeks. Broadly, stillbirth is classified into antepartum and intrapartum stillbirths based on whether the fetal death occurs before or after the onset of labor [2]. Most stillbirths occur in marginalized populations. For example, in high-income countries, women belonging to low social classes exhibit double the risk of stillbirths as compared to economically better-off women [3, 4]. A meta-analysis has shown that the magnitude of stillbirth remains high in low-middle-income countries (LMICs) and that associated factors vary across the countries and include maternal illiteracy, poverty, lack of antenatal care, extremes of maternal ages, and previous history of stillbirth [5].

The ‘Every Newborn Action Plan (ENAP)‘has set a target of < 12 stillbirths per 1000 births to be achieved by 2030 by every country [6]. High and upper-middle-income countries have already achieved this target; however, it may be unachievable for low and middle-income countries.

In Pakistan, despite numerous initiatives for safe motherhood such as formation of Maternal, Newborn, and Child Health (MNCH) facilities for childbirth and initiation of a community midwife (CMW) program to facilitate community-based deliveries, no substantial decline has been observed either in maternal mortality or in stillbirths; the stillbirth rates remain high at 43 per 1000 births [7, 8]. The urban-rural difference in home-based deliveries is highest in the Sindh province of Pakistan with 22% of births occurring at home in urban areas as compared to 53% in rural areas with resulting high numbers of maternal complications and stillbirth in the later [9]. To the best of our knowledge, very little or no information is available on women’s perspective regarding causes of stillbirths.

**Fig. 1:**
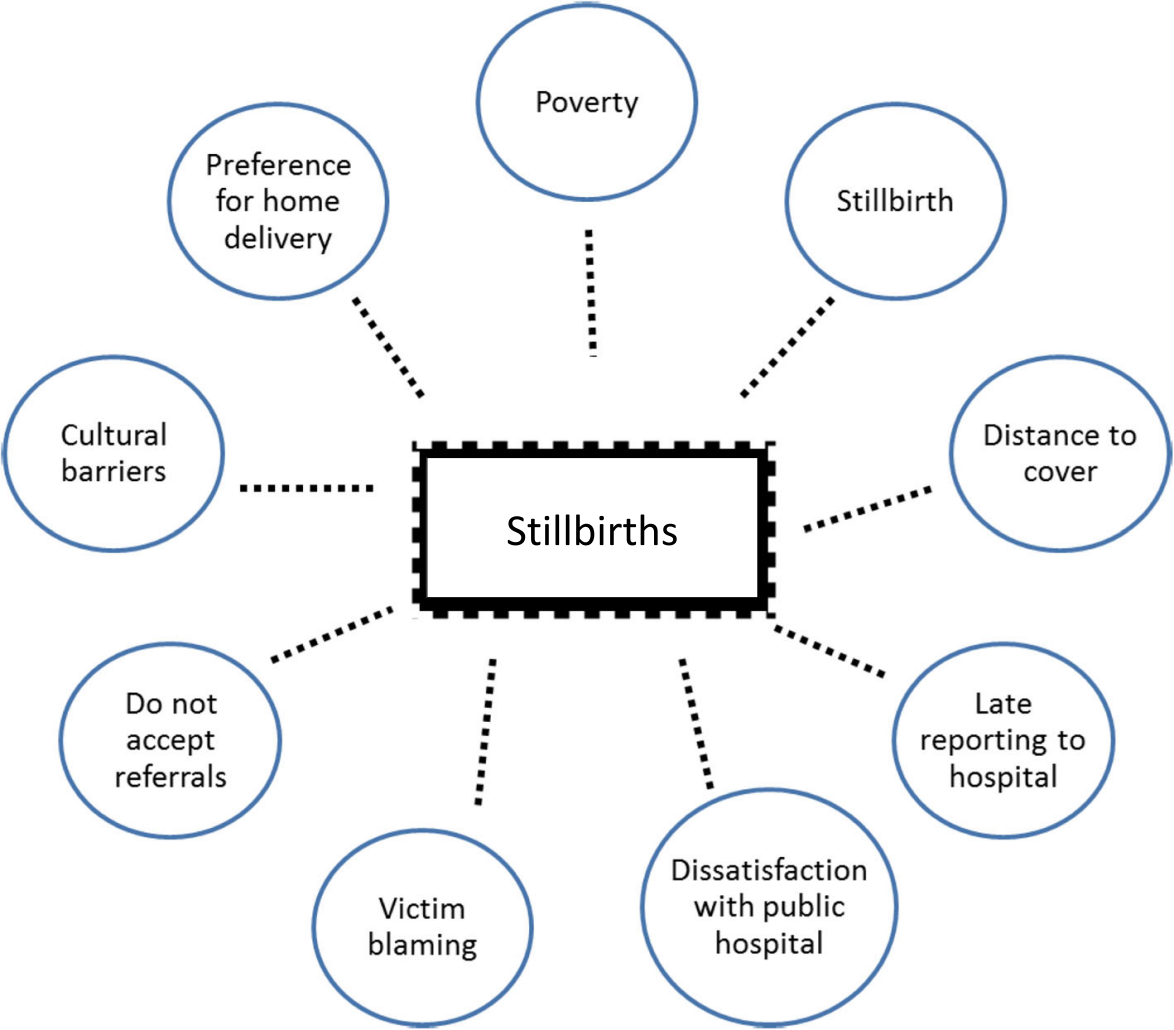
Conceptual Framework for high stillbirths in Thatta District. The Global Network for Women’s and Children’s Health maintain a Maternal and Newborn Health Registry (MNHR) in 14 geographically defined study clusters of Thatta district. This is a prospective, population-based observational study that includes all pregnant women and their outcomes [10]

The conceptual framework, which we followed, was based on the facts established on our experience of working in Thatta and from the MNHR database. Based on this experience, we hypothesized that the high occurrence of stillbirths in Thatta is due to a number of factors where main drivers towards stillbirth are poverty which hinders women from seeking hospital delivery, inability to recognize danger signs in pregnancy and lastly cultural barriers and past unpleasant experiences of hospital delivery which may push women to seek home delivery by an unskilled health care provider. (Fig. 1).

Registry administrators (RAs) are paid community health workers or nurses who identify pregnant women in their respective areas and after consent, enroll them in the MNHR. Once a pregnant woman is identified, the RAs obtain basic health information; record the date of the last menstrual period or early ultrasound report to assess gestational age. A follow-up visit is carried out following delivery to collect information on pregnancy outcomes and health care received during delivery. According to this active surveillance, the study clusters at Thatta, show high rates of stillbirth ranging from 58.4/1000 births in 2010 to 48/1000 births in 2016 with an average annual reduction rate of 2.4% [6, 11]..

The study results provide information for researchers, policy makers and public health institutions to guide and enhance efforts for prevention of high stillbirths in Thatta and other rural areas.

## Methods

### Study setting

The study was conducted in Rural District Thatta of Sindh Province during June 2017-Sept 2017 for master’s thesis of Health Policy and Management Degree requirement by the first author. District Thatta has a population of one million with a reasonable infrastructure of public and private health facilities providing pregnancy and delivery care. There is one District Health Hospital (DH), one sub-district level hospital, 6 rural health centers, 22 basic health units, 10 dispensaries and a number of private facilities. This study was conducted in the catchment population of Global Network Birth Registry, which has a population of 180,000 [12].

### Study design and study participants

We used an exploratory qualitative study design by conducting in-depth interviews (IDIs) and focus group discussions (FGDs) using a semi-structured interview guide. Four IDIs were conducted with women who had experienced a stillbirth. Four FGDs were conducted with married rural women (FGDs = 4; *n* = 29); three FGDs included 7 rural women; and one FGD included 8 rural women. FGDs were also conducted with traditional birth attendants (TBAs) (FGDs = 4; *n* = 14); two FGDs included 4 TBAs and two FGDs included 3 TBAs. Married women irrespective of experiencing stillbirth were interviewed to understand community’s practices related to antepartum and intra-partum care and stillbirths. TBAs were interviewed to understand their practices related to stillbirth and to see any relationship with women’s perceptions. In-depth interviews with women who experienced stillbirth were conducted to understand the issues resulting in fetal death. The eligibility criteria for in-depth interviews was to include women who experienced stillbirth within last 3 months of the date of delivery interview regardless of place of delivery; for focus group discussions with rural women included women who experienced pregnancy during past 2 years of date of the interview irrespective of pregnancy outcome; TBAs who had more than 5 years of experience of conducting deliveries in Thatta and in practice; study participants to be permanent residents of Thatta. All the participants were identified through purposive sampling with the help of the Global Network, MNHR database. TBAs were identified through locally maintained database at research office of Thatta which is not part of the MNHR.

### Interview guides

Semi-structured data collection tools were developed in English language, which were translated into local language Sindhi to capture perceptions of the study participants. The interview guide for women included question on overall pregnancy experience, support system, decision making for place of delivery, health seeking behavior, perceptions regarding stillbirths, reasons for high stillbirths, challenges faced during pregnancy, and views about maternal healthcare services offered at health facilities. The interview guide for TBAs included questions on their understanding and knowledge of stillbirths, barriers and challenges faced by them for providing care during perinatal period, prevention of stillbirths, home based versus facility based deliveries, and quality of delivery services. The interviews were conducted after taking written informed consent from each participant. The FDGs and IDIs were carried out until the data became saturated and no new information emerged. All the interviews were carried out in the local language Sindhi. The lead author was fluent in local language and carried out all the interviews with two research assistants with Master’s degree and specially trained for this research.

### Ethical approval

The Ethics Review Committee of Aga Khan University, Karachi, Pakistan, gave the ethical approval for the study.

### Data analysis

The data were transcribed from Sindhi to English and were analyzed manually. Qualitative content analysis was conducted to understand the manifest content (what the transcript states) and latent content (understanding of the meaning of the transcript). Transcriptions were made from all the voice recorded data. Transcripts were read various times by two independent investigators to develop an interpretation of the participants’ perception regarding high rates of stillbirths. The text was divided into ‘meaning units’ that was shortened and labeled with a ‘code’ with mutual agreement between the two individuals without losing the study context. Codes were then analyzed and assembled into categories to capture the manifest meaning. In the final step, themes and subthemes were identified. Study rigor was ensured by triangulation of different data sources (rural women, TBAs) and data collection methods (FGDs and IDIs).

## Results

Four IDIs and eight FGDs were conducted. Table 1 describes the demographic characteristics of study participants. Most of the women from the communities were illiterate, and few of them had completed primary education. All women were between the ages of 23 to 39 years. The parity of women ranged from 01 to 08. Almost all TBAs were illiterate, and only one could read and write without receiving formal education. All TBAs aged between 45 and 60 years (Table 2). Average numbers of deliveries performed by each TBA per month was 4–5.

**Table 1:** Demographic details of the community participants and traditional birth attendants (TBAs)

Data collection method	Age range	Education	Parity	Experienced IUD in previous pregnancies	Place of delivery and stillbirth category
Four IDIs with women who experienced stillbirths (IDIs = 4)	23–36	Illiterate (2) Primary (1) Class 10 (1)	1 (1) 2 (1) 4 (1) 8 (1)	All women experienced Stillbirths	Antepartum stillbirth (3) Intra-partum stillbirths (1) Home delivery (1) Facility Delivery (3)
Four FGDs with rural women in general (FGDs = 4;*n* = 29) Three FGDs included 7 rural women; and one FGD included 8 rural women	23–39	Illiterate (18) Primary (5) Class 10 (4) Intermediate^a^ (1) BA^b^ (1)	1–3 (12) 4–5 (8) 5–7 (6) 8 and more (3)	IUD^c^ (6) One woman reported having 3 IUDs	NA
Sample size	Age	Education	Position/post	Working experience	
Four FGDs with Traditional birth attendants (FGDs = 4;n = 14) Two FGDs included 4 TBAs and two FGDs included 3 TBAs	45–60	Illiterate (13) Informal training (1)	Conduct home deliveries in villages	Average number of deliveries performed per month 1–4 (10) 5–7 (4)	

^a^Class 12th

^b^Bachelors of Arts

^c^Intrauterine device

**Table 2:** Characteristics of birth attendants and key informants

Sample size	Age	Education	Position/post	Working experience
Four FGDs with Traditional birth attendants (14)	45–60	Illiterate (13) Informal training (1)	Conduct home deliveries in villages	Average number of deliveries performed per month 1–4 (10) 5–7 (4)
2 Skilled birth attendants 1 KIIs with CMW^a^ and 1 with LHV	CMW 26 LHV^b^ 55	Primary (LHV) Class 10th (CMW)	CMW 18 months LHV 19 years (conducted delivery for last 5 years)	CMW = 18 months LHV = 19 years
2 obstetricians One from public sector One from private sector	40–60	MBBS^c^; FCPS^d^ Obstetrician	One obstetrician is HOD^e^ of Obs/gyne department of District Hospital Thatta. Private practitioner owns secondary care hospital	29 years of experience HOD of District Hospital. 9 years of experience

^a^Community midwives

^b^Lady Health Visitor

^c^Bachelor of Medicine, Bachelor of Surgery

^d^Fellowship of the College of Physicians and Surgeons

^e^Head of Department

Two major themes for high stillbirths in Thatta emerged from the perspectives of women and TBAs: (1) factors influencing health seeking behavior of women during pregnancy and childbirth and, (2) perceived barriers and challenges in accessing pregnancy and delivery care.

### Factors influencing health seeking behavior of women during pregnancy and childbirth

Health seeking behavior of women in Thatta showed very interesting but alarming pattern. They have developed their own understanding about adjudicating the severity of the condition during pregnancy and childbirth and for seeking medical help. Based on these perceptions they would decide to consult a health care provider, follow or ignore the advice of the health care provider, or not to seek help at all. These decision-making processes are deeply rooted in past experiences, resources to afford the cost of treatment, cultural practices and prevailing myths, and the wisdom of elderly women. For example, in almost all the FGDs, women mentioned that they seek ANC from a health facility mostly in their first and second trimester to confirm pregnancy either through pregnancy test or through ultrasound as these are the most confirmatory sources but not thereafter. In the last days of pregnancy, they visit community TBAs to confirm the cephalic position of the fetus or for external cephalic version of the fetus if needed. This was confirmed by TBAs too during FGDs as well.

*“Women of our village come to us for abdominal massage in late pregnancy to position the head of the baby down for normal vaginal delivery” (TBAs – FGDs)*

To continue to seek routine ANC is considered expensive, time consuming and not needed. Confirmation of pregnancy and later vertex position of the baby is considered as sufficient information and reassurance for a normal vaginal delivery.

Most of the women considered fever, odor from vaginal secretions or signs of any other illness during pregnancy as trivial and consider that these either would resolve on their own or would take home remedies to treat these. This was reflected in one of the in-depth interviews where a woman who recently had a stillbirth, mentioned that she had foul smelling vaginal discharge during pregnancy, for which she consulted a physician who advised her some laboratory tests and prescribed her medications. She did not follow this advice;

“*I did not go for lab tests which a doctor had recommended. I thought fever and lower abdominal pain would subside by taking rest*.” *(Woman who had stillbirth - IDI)*

The woman lost her baby, the reasons again being trivial response to illness and seeking care. In some cases poverty may force family to leave the situation to faith. As One TBA during FGD mentioned her experience saying; “*… when I went to see the mother, she was having high grade fever, foul smell discharge from vagina and her baby was also dead in the uterus. … and she was having severe infection … I asked her husband to take her to hospital but he denied and later the mother died.” (TBA-FGDs).* Husband could not afford the cost of travel, admission to the hospital and treatment.

The wisdom of elderly females in the family may not allow younger females to follow the advice given by the skilled health care providers. For example, women who seek advice from some health care provider in pregnancy and receive nutritional supplements are discouraged by the elderly family members in the family to use it on the pretext that supplements would increase the weight of the baby which would result in a delivery through an operation (caesarian section). One of the women, who had a stillbirth, during her in-depth interviews shared that she was 6 months pregnant and had anemia. A physician advised her to take nutrition supplement, however her mother-in law did not allow her to take the supplement

*“My mother-in-law told me that these supplements will make my baby big which will result in difficulty in normal delivery and might have an operation (C-section)” (Woman experienced stillbirth - IDI)*

This fear of operative delivery (C. section) came under discussion at length in one of the focus group discussions as well. Women expressed their preference for having home delivery by a TBA over a facility-based delivery even when facing a complication due to fear of delivering through C. section. They thought doctors conduct unnecessary operations for earning money.

*“In hospitals, doctors don’t assess women properly and say that the baby needs to be delivered through an operation … ” (Woman - FGDs)*

FGDs revealed that women are under social pressure to conceive soon after a stillbirth as they might not conceive again. Also they considered using any family planning method after stillbirth weakens the uterus resulting in miscarriages. One woman commented;

*“ … my friend’s uterus got weak after using some method of family planning after stillbirth; that’s why her later pregnancies ended up in 7 or 8 months of pregnancy” (Woman - FGD)*

This belief is supported by the TBAs

*“We place indigenous medicine in woman’s uterus after a macerated stillbirth to remove poison from her body … and advise her to conceive soon.” (TBAs - FGDs)*

### Perceived barriers and challenges in accessing pregnancy and delivery care

In nearly all the focus group discussions, women mentioned difficulty in accessing care due to non-availability of female doctors, high cost of care, far distance to facility, lack of transport and need of an escort from family or village to visit a health facility. Women mentioned that it is easier for them to access a traditional birth attendant who lives in the same village and is easily available, her service charges are nominal or is satisfied if paid in kind. In some areas of Thatta non-availability of female doctors force women to seek care from unskilled birth attendant as going to far-off areas gets costly. These are probably the driving force for women to ignore medical advice or avoid routine care during pregnancy and childbirth as mentioned above.

*“Here in Jhangshahi we don’t have female doctors, our pregnant women have to travel to the city for antenatal care and everybody cannot afford to go to the city.” (Woman - FGDs)*

Word of mouth plays an important role in rural communities to base their actions on the positive or negative experiences, which the other women had experienced at a health care facility. This factor was highlighted in the discussions where some women who had the experience of receiving care from the skilled birth attendants such as doctors, nurses and midwives at public or private health facilities. They mentioned that skilled birth attendants in their clinics lack essential medical supplies such as instruments to feel fetal heart beat (fetoscopes or Doppler) for the assessment of baby’s condition during antenatal and delivery care and non-functioning of the necessary equipment at the facilities such as blood pressure apparatuses.

*“We know that in every antenatal visit, blood pressure should be checked. However, for most of the time, BP machines are not working” (Women - FGDs).*

They complained that doctors do not give sufficient time and information to them on their or the baby’s health status, so they remained unaware of the condition, which lead to delay in seeking care. Hence contacting a TBA is easier and cheaper, who is available and gives time.

Women also mentioned that at government clinics and hospitals, ultrasound examination is provided only once a week, and women with any warning signs during pregnancy which require an ultrasound examination have to wait for a whole week to get their ultrasound done.

*“Poor people can’t afford private clinics; therefore, government hospitals are the only option. At the district hospital, ultrasound is provided only once a week, so we have to wait for that long.” (Women - FDGs)*

*“On my regular antenatal visit, the doctor gave me some medicines and asked me to come back after a week. One week later she asked for an ultrasound and informed me that my baby has died” (Woman who experienced a stillbirth - IDI)*

Women in focus group discussions mentioned that even when they reach the hospital in time, their babies might not survive. Only the district hospital of Thatta accepts complicated cases but due to poor treatment facilities and non-availability of staff, the outcome is mostly poor. One of the women who had a stillbirth said;

*“A private doctor informed me that my baby’s heartbeat is getting slow and referred me to the district hospital for delivery, there I remained admitted for one whole day but no one even checked my blood pressure, when I started to get seizures then they simply referred me to a hospital in Hyderabad (city)”(The woman experienced Stillbirth - IDI)*

*“Doctors are not present on their duties … when a woman’s condition gets serious then either they run to give treatment or refer her to another hospital” (Woman- IDI)*

TBAs also expressed their limitations for conducting home deliveries in villages. They find difficulty in following the standard practice of delivery care due to lack of skills and non-availability of resources for identification of fetal distress. TBAs further mentioned that for resuscitation of newborn for home-based deliveries they lack essential skills and equipment. Few TBAs use either mouth breathing or traditional practices to resuscitate apparently stillbirth infants.

*“ … If a baby does not breathe after delivery we give mouth to mouth breathing using a piece of cloth...if we don’t give our breath to the baby it will not survive” (TBAs-FGDs)*

*“ … We help babies breathe by heating placenta on a steel plate (Tawa)” (TBA - FGD)*

*“If a baby has difficulty in breathing, we refer the case to nearby clinics, but these babies are considered dead and are not treated and referred by the health care provider to the district hospital” (TBA - FDGs)*

TBAs discussed that previously they were given delivery Kits free of cost by the government or NGO resources to conduct safe deliveries. Now they lack supplies therefore for un known reasons and they have to conduct delivery through unsafe techniques such as not wearing gloves. Further, they shared that they need government support so that they can identify women at early stage of pregnancy complications and can contribute to prevent stillbirths.

*“ … we are not getting any support from the government like provision of safe delivery kits to practices safe deliveries at home--- we have buy new gloves for delivery ourselves” (TBA in FDG)*

## Discussion

This study provided a unique opportunity to understand perceptions and experiences of rural women and TBAs regarding stillbirths. Women mentioned their apprehension for operative delivery, lack of compliance with the treatment, difficulty in accessing care, high cost of care, far distance to facility, lack of transport and need of an escort from family or her village to visit a health facility. The distance to the facility and poverty make women ignore danger signs of pregnancy on the hope that they would subside on their own, this attitude may lead to harmful behavior [5].. The easy availability of unskilled providers in the form of traditional birth attendants is then preferred over a skilled health care provider who may be too far to reach and expensive. Numerous studies showings that deliveries conducted by C. section in the hospitals add to the cost and duration of stay in the hospital adding to the fear against hospital deliveries [13]. Besides, there is a need to raise the awareness of villagers and about the danger signs of pregnancy and delivery, and how to monitor the fetal well-being [14, 15]. Community midwives, LHVs, and LHWs could give these awareness trainings to rural women and TBAs through regular community-based group sessions. Women need assurance that C-section is performed to save the life of the fetus before it is too late. Some social safety nets to cover the costs of delivery care are required for poor populations. Improving the quality of care at the time of birth in public hospitals can address many of these issues and will go a long way to build the trust of women on facility delivery [16]. Moreover this study results added another important issue raised by women was lack of functional equipment in the public facilities. Simple equipment such as blood pressure apparatuses and Doppler machines to monitor fetal well-being are a necessity and their maintenance, or replacement is the responsibility of the hospital administration of both public and private health facilities. The lack of basic equipment should never be the excuse for the poor provision of care, especially in public hospitals.

The Government of Pakistan has recently established a health Care Commission in every province. It is an autonomous regulatory authority to ensure quality of healthcare in all the provinces through implementation of Minimum Service Delivery Standards (MSDS) in both public and private sector healthcare establishments [17]. This initiative would help both patients and those providing healthcare facilities by addressing issues of medical negligence, maladministration and malpractice. Only time will show how this would impact the outcome on maternal and fetal mortality.

Moreover, similar findings shown in other studies that poor quality of care and lack of skills to resuscitate newborns who do not show signs of life at birth were important factors to consider at the facility and health care provider’s level [18, 19]. The government of Pakistan has introduced many programs with trainings on technical skill building, program management and data management, etc. [20]. However, many of these initiatives did not address the trainings on professionalism and work ethics for the service providers. In most rural areas, populations are illiterate, very poor and highly vulnerable, and they suffer because of the poor behavior and attitude of the providers [18]. Respectful maternity care should become the norm especially for public health facilities providing pregnancy and delivery care.

A substantial number of deliveries occur at home in rural areas by TBAs. Few studies reported the similar findings that deployment of community midwives at villages for safe home deliveries and linkages of local ambulance services with midwives and communities to facilitate the timely transfer of women in labor to a higher level of care can help in reducing high maternal mortality and stillbirth in Thatta [21–25]. Meanwhile, unskilled birth attendants need support, monitoring and trainings until skilled care is available to each and every pregnant woman in Thatta and Pakistan.

Women and TBAs who were living in marginalized areas were unable to reached and not included in the study. We believe that their perceptions regarding high rates of stillbirth in Thatta might enhance the results of this study.

**Fig. 2:**
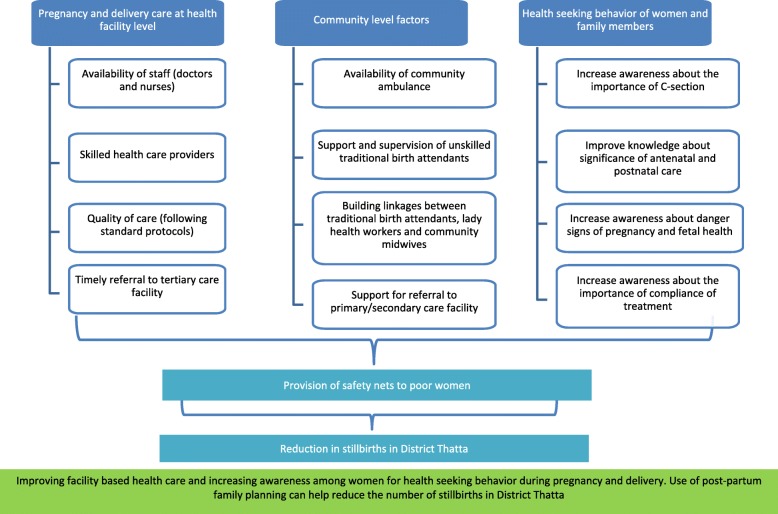
Proposed framework for reducing stillbirths in Thatta District

Based on the results of these perceptions we propose a framework for the researchers, policy makers and public health institutions to guide and enhance efforts for prevention of stillbirths in Thatta and similar areas (Fig. 2). This framework focuses on three main levels of health system including health facility level, community level, and individual level. The health facility level domain emphasize mainly on requirements needed to provide quality healthcare services such as, availability of trained health care providers (doctors, nurses), standard protocols, and timely referrals to tertiary healthcare facility. The second domain involves community level factors which include availability of equipped ambulance, supervision of TBAs, support for referral to primary and secondary healthcare facilities, and effective linkages between TBAs and CMWs and LHWs. The third domain mainly involves individual level factors such as, health seeking behaviors of women, awareness about the danger signs of pregnancy and fetal health, and knowledge about the importance of antenatal and postnatal care, and C-section. Continuous efforts on all three domains would ensure provision of safety nets to poor women which will eventually reduce the rate of stillbirths in district Thatta.

In conclusion, study results emerges two major theme to understand the high rates of stillbirths in Thatta. First, factors influencing health seeking behavior of women during pregnancy and childbirth and second, perceived barriers and challenges in accessing pregnancy and delivery care. Hence, conscious efforts are required to increase the awareness of women for positive health seeking behavior during pregnancy, delivery and post-partum period. Skills and professional behavior of health care providers also need to be enhanced for provision of quality of care especially for respectful maternity care.

## Data Availability

All data generated or analyzed during this current study available from the corresponding author on reasonable request.

## Abbreviations

CMW: Community midwife
DH: District Health Hospital
ENAP: Every Newborn Action Plan
ERC: Ethical Review Committee
FGDs: Focus group discussions
IDIs: In-depth interviews
LHVs: Lady Health Visitors
LMIC: Low-and-middle-income-countries
MNCH: Maternal, Newborn, and Child Health
MNHR: Maternal and Newborn Health Registry
RAs: Registry administrators
TBAs: traditional birth attendants
WHO: World Health Organization
